# Mapping of QTL affecting incidence of blood and meat inclusions in egg layers

**DOI:** 10.1186/1471-2156-12-55

**Published:** 2011-06-13

**Authors:** Mervi Honkatukia, Maria Tuiskula-Haavisto, Virpi Ahola, Pekka Uimari, Matthias Schmutz, Rudolf Preisinger, David Cavero, Pia Vennerström, Jesus Arango, Neil O'Sullivan, Janet Fulton, Johanna Vilkki

**Affiliations:** 1 Biotechnology and Food Research, MTT, Jokioinen, 31600, Finland; 2 Department of Biosciences, University of Helsinki, Helsinki, 00014, Finland; 3 Lohmann Tierzucht GmbH, Cuxhaven, 27472, Germany; 4 Research Department, Fish and Wildlife Health, EVIRA, Mustialankatu 3, Helsinki, 00790, Finland; 5 Hy-Line International, P.O. Box 310, Dallas Center, IA 50063, USA

## Abstract

**Background:**

Occurrence of blood and meat inclusions is an internal egg quality defect. Mass candling reveals most of the spots, but because brown eggshell hampers selection in brown chicken lines it has not been possible to eliminate the defect by selection. Estimated frequency of blood and meat inclusions in brown layers is about 18% whereas it is 0.5% in white egg layers. Several factors are known to increase the incidence of this fault: genetic background, low level of vitamin A and/or D, stress or infections, for instance. To study the genetic background of the defect, a mapping population of 1599 F_2 _hens from a cross of White Rock and Rhode Island Red lines was set up.

**Results:**

Our histopathological analyses show that blood spots consist of mainly erythrocytes and that meat spots are accumulations of necrotic material. Linkage analysis of 27 chromosomes with 162 microsatellite markers revealed one significant quantitative trait locus (QTL) affecting blood spot and meat spot frequency. We sequenced a fragment of a candidate gene within the region, *ZO-2*, coding for a tight junction protein. Nine polymorphisms were detected and two of them were included in fine-mapping and association analysis. Fine-mapping defined the QTL result. To further verify the QTL, association analyses were carried out in two independent commercial breeding lines with the marker MCW241 and surrounding SNPs. Association was found mainly in a 0.8 Mb-wide chromosomal area on GGAZ.

**Conclusions:**

There was good agreement between the location of the QTL region on chromosome Z and the association results in the commercial breeds analyzed. Variations found in *tight junction protein ZO-2 *and microRNA *gga-mir-1556 *may predispose egg layers to blood and meat spot defects. This paper describes the first results of detailed QTL analyses of the blood and meat spots trait(s) in chickens.

## Background

Egg quality has received more attention due to increased demands for safety and high-quality eggs by consumers. Internal egg quality involves functional, aesthetic and microbiological properties of the egg yolk and albumen. Internal inclusions (blood and meat spots) in the egg have been recognized as quality defects since 1899 [1]. In addition to being an aesthetic and ethical problem, there is indication that blood or pieces of tissue inside the egg may increase the risk of infections such as salmonella [2] and reduce hatchability of eggs [3].

Blood spots are droplets of blood found usually on the surface of the yolk [4,5]. Meat spots appear as red, brown or white spots in the albumen. They are either pieces of tissue from reproductive organs or blood spots that have changed colour due to dilution. The factors causing inclusions are unknown. They emerge during the ovulation process in the ovary or later in the oviduct. Blood on the yolk originates from bleeding of the small vessels in the ovary or in the oviduct [5]. When blood is found adhering to the yolk, bleeding has occurred in the ovary at the time when the yolk was released from the follicle. The follicle has a dense network of blood vessels, aside from an avascular area of the follicular wall, called the stigma. The follicle sac ruptures at the stigma during ovulation. If any blood vessels cross the stigma, a small drop of blood may be deposited on the yolk as it is released from the follicle. Alternatively, bleeding may occur before ovulation on the vitelline membrane, the structure directly adjacent to the outer surface of the yolk. In that case, blood spots are found in the space between the follicular wall and the vitelline membrane [4]. Blood in the albumen indicates bleeding shortly after the release of the yolk into the oviduct, at the time when the yolk is coated with albumen. Meat spots in the albumen can be formed from a bit of reproductive tissue while the egg is passing through the oviduct. As an egg ages, the yolk takes up water from the albumen, which in turn dilutes blood spots and makes them look like meat spots.

In general the frequency of blood and meat inclusions is less than 1% in all eggs laid in present commercial lines [2]. However, the incidence of spots varies greatly: it is about 18% in brown layers, whereas it is only 0.5% in white egg layers [6]. In some brown layer lines the frequency can be as high as 30% [6]. The incidence of spots seems to increase when the hen ages [7]. Increased frequency also appears at the start of laying.

Different types of factors, including nutritional, environmental and hereditary factors, trigger the incidence of spots. Probably the most important nutritional factor is a lack of vitamins A and D [3, 8, 9, 10]. There is a physiological threshold for the amount of vitamin A. When the supply is sufficient, the chicken has a low probability of having blood spots [11]. Environmental factors, like sudden loud noises, temperature changes and infections, induce an increase in the incidence of spots [4,6,12]. Furthermore, the problem has a genetic background. The estimates of heritability for inclusion traits range from 0.07 up to 0.6 [11,13,14]. In conventional breeding practices, selection against inclusions is usually done by eliminating families that have increased incidence of inclusions from the breeding population (Schmutz, M., personal communication).

Internal inclusions are detected via mass candling. This process reveals most of the spots, but occasionally faulty eggs pass the control checks and end up in the consumer's hands. This has been a problem especially in brown layer lines, because brown shells hamper spot detection.

Increased interest in safety and high-quality eggs motivated us to study the genetic background of the incidence of blood spots in chicken eggs. So far, there have been no attempts to map QTL for internal inclusions. The ultimate objective was to find markers suitable for use in a commercial selection programme and to identify genes affecting the defect.

## Results

### Histopathologic study

In the analyzed broiler eggs, the spots that were collected close to the surface of the yolk, macroscopically looking like blood stains, were accumulations of erythrocytes surrounded by a thin eosinophilic acellular membrane. Spots found in the albumen, macroscopically looking like a greyish mass, were accumulations of necrotic material surrounded by a thin acellular membrane. The necrotic material consisted of fine granular eosinophilic and brown debris.

### Genetic parameters, heritabilities and genetic correlations

The heritability estimates in pure lines for blood spot score and blood spot combination (number*size) were 0.05 and 0.04, respectively, in Lohmann Brown. In White Rock the heritability of blood spot score was 0.01. The heritability for meat spot combination (number*size) was evaluated to be 0.01 in Lohmann Brown (no data available for White Rock). The genetic correlation between inclusion traits varied greatly: it was highly negative (-0.90) between two blood spot traits (score and combination) and between blood spot score and meat spot combination (-0.70), whereas it was positive (0.83) between blood spot combination and meat spot combination (see additional file 1, Table S1).

### Genome scan

One genome-wide significant QTL affecting incidence of internal inclusions was discovered (Table 1). The highest F-ratio, 18.59, occurred at 69 cM, in the area flanked by markers MCW258 and MCW241 (21,403,330-34,264,330 Mb) on chromosome Z. The additive effect was 3.61 with 0.84 SE, corresponding to a difference of 0.036 in the average score from three consecutive eggs (this trait has an average of 1.02, with a standard deviation of 0.24). It explains 1% of the total phenotypic variance.

**Table 1:** Summary of QTL results for blood and meat spot trait (BMS_F2_) from the F_2 _genome scan

**Chrom**.	pos cM	Flanking markers and genomic positions	F^(1)^	1%^(2)^	5%^(2)^	10%^(2)^	Add.^(4)^	SE^(5)^	Dom.^(4)^	SE	R^2(3)^
1	429	MCW0023-MCW0145 156,472,062 - 162,032,735	5,86	8,64	7,52	4,33	-2,27	1,48	7,07	2,35	2%
2	0	MCW0082 5,313,874	6,90	10,90	8,60	4,90	-4,32	1,43	4,38	2,19	2%
4	113	MCW0284-ADL331 54,907,139 - 63,195,046	6,25	10,33	8,00	5,18	-3,64	1,30	-3,87	1,90	2%
Z	69	MCW258-MCW241 21,403,330 - 34,264,059	18,59	16,24	13,63	12,2	3,61	0,84	na	na	1%

The effect was calculated as the effect of the allele originating from the parental RIR line. The marker map used in the Z-chromosome was: ADL117-(22cM)-MCW331-(12 cM)-MCW55-(16cM)-MCW258-(38cM)-MCW241.

^1 ^F-ratio for the regression analysis.

^2 ^Genome wide significant levels

^3 ^R^2 ^is the reduction (in %) of total phenotypic variance due to the presence of the QTL.

**^4 ^**Real effect is the value divided by 100 (Add = additive effect, Dom = dominance effect)

^5 ^SE = standard error

Other suggestive (genome-wide 10% significance) QTL were found on chromosome 1 at the position of 429 cM between markers MCW23 and MCW145 (156,472,062-162,032,936 Mb), on chromosome 2 at the beginning of the linkage map at the marker MCW82 (5,313,874-5,313,971 Mb), and on chromosome 4 at the marker ADL331 (63,195,046-63,195,223 Mb). The QTL effect on chromosome 1 is dominant while it is additive for the other two regions.

### Sequencing of *ZO-2*

**Figure 1:**
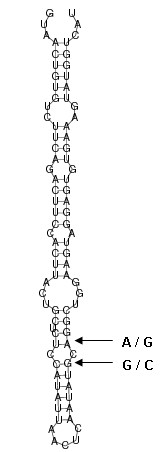
**Hairpin structure of MI0007281 (mir-gga-1556)**. The prediction of the structure is constructed with RNAfold web server. Variations found by sequencing are indicated with arrows.

A putative candidate gene, *ZO-2 *(NP_990249), a key gene controlling adhesion between neighbouring epithelial cells, was found to be located within the QTL region on chromosome Z. The marker MCW241 showing significant association to blood and meat inclusions was located inside this gene. We sequenced a fragment of 549 bp from several individuals from this candidate gene among Lohmann Brown and Hy-Line populations (106 and 20, respectively). We discovered nine polymorphic sites (Table 2). Three of the variations were located in exon 18. One of the previously reported SNPs, rs10724503 http://www.ensembl.org/Gallus_gallus/ was confirmed and used as a marker (ZO-2 snupe) in the following association study. The other reported frameshift mutation rs16767170 http://www.ensembl.org/Gallus_gallus/) was not detected among this material. Two new intronic variations (SNP6, position 34,315,888 and SNP7, position 34,315, 890 in Table 2) were found to be located within a microRNA (*gga-mir-1556*). These polymorphisms were located in the predicted stem-loop structure of the microRNA (Figure 1). Such variation might affect the stability of the stem-loop structure and thus also the function of the miRNA in gene regulation. One of these variations was used in the association study (SNP7, 'miRNA'). The variations have been submitted to GeneBank (BankIt 1438826, JF509397).

**Table 2:** Variation found in the *ZO-2 *gene

SNP #	genomic location	exon/intron	additional information	flanking sequence
**1**	34,315,482	intron17-18		TCTGAATGCA**[A/G]**TATAACTGTA

**2**	34,315,545	intron17-18		ATAAGATGTT**[C/T]**CACACCCTGC

**3**	34,315,708	exon18	rs10724503	GTAAGCAGGG**[C/T]**GTGAAAACGA

**4**	34,315,757	exon18		AAAAGCTCGA**[A/G]**GAAGCTTTAT

**5**	34,315,792	exon18		AGCTGAAGAA**[A/G]**ACTTGTTCCC

**6**	34,315,888	intron18-19	gga-mir-1556	TTACTGCTCT**[C/G]**CGTATTAACT

**7**	34,315,890	intron18-19	gga-mir-1556	ACTGCTCTGC**[G/A]**TATTAACTCA

**8**	34,315,932	intron18-19		GTGTGAAAGT**[A/G]**TGGTCATGAG

**9**	34,315,953	intron18-19		CAATCTCTGA**[C/T]**TTCCTCTCAA

Locations of polymorphisms are shown together with the flanking sequences.

### Fine-mapping of chromosome Z

**Figure 2:**
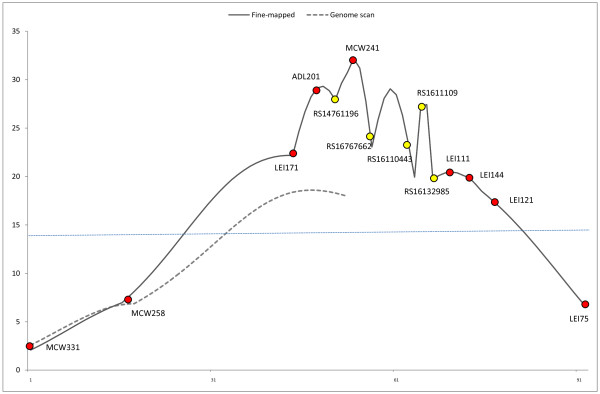
**QTL analysis of the blood and meat spot trait (BMS_F2_) in the F_2 _mapping population**. The F-ratio curves from the initial genome scan (dashed line; 7 families, 5 microsatellite markers) and the fine-mapping stage (solid line; 17 families, 9 microsatellite markers, 5 SNPs) are shown. The genome-wide significance level is marked as a dotted line. The microsatellite marker positions (fine-mapping stage) are indicated with red circles and the SNP positions with yellow circles in the following order: MCW331, MCW258, LEI171, ADL201, rs14761196, MCW241, rs16767662, rs16110443, rs1611109, rs16132985, LEI111, LEI144, LEI121, LEI75.

When a larger sample of animals was genotyped for a dense map of the QTL region on chromosome Z, a genome-wide highly significant QTL (F-ratio 32.9) was seen at the marker position MCW241, at the position 54 cM on the linkage map (genomic location Z: 34.26 Mb) with the additive effect of 3.75 units (Figure 2). Compared to the initial QTL scan, more markers were added to the distal end of the map to flank the QTL. As a result, the highest peak for F-ratio shifted outside of the previously mapped area, but it was successfully flanked with new markers. The effect of the QTL was now estimated to be 2% of the phenotypic variance.

### Association

Association was studied with a set of SNP markers (including miRNA variation) and the most significant microsatellite marker MCW241 (Table 3). The studied SNP markers were located between bp 31,855,282 and 36,533,455 of chromosome Z. Associations were found mainly in a 0.8 Mb-wide chromosomal area located between 33,508,907 - 34,315,890 of GGAZ (Table 3). The associated loci varied according to the studied phenotype and population. For instance, in the Hy-Line population only the meat spot trait was found to be associated to SNP 'rs14761267', whereas SNP 'rs14761196' was linked to both the meat and blood spot traits. Most of the associations were related to the prevalence of the spots (score or number of the spots). The allelic effects varied between 0.13 and 0.5 SD, depending on both trait and population.

**Table 3:** Association of markers with different internal inclusion traits in the confirmation study

SNP ID/marker	Genomic position	Marker informative in	Association found in	Trait and p-values
rs14762832	31,855,282	LB	LB	Score _LB _(p < 0.001)

rs16766794	31,955,874	LB		

rs16766752	32,044,210	LB		

rs13795687	32,122,845	LB		

rs16766685	32,276,606	LB		

rs16766334	33,022,548	LB		score _LB _(p < 0.05)

rs16766274	33,092,178	LB		

rs16766257	33,167,074	LB		

rs14761556	33,443,086	LB		

rs14761487	33,508,907	LB	LB	score_LB _(p < 0.01) group_LB _(p = 0.02) number of spots_LB _(p < 0.05)

rs14761341	33,749,060	LB, Hy		

rs14761267	33,832,110	LB, Hy	LB, Hy	number_LB _(p < 0.02) size _LB _(p < 0.02) MS_Hy _(p < 0.05)

rs14761196	33,996,581	Hy	Hy	MS_Hy _(p < 0.01) BL_Hy _(p < 0.04)

MCW241	34,264,059	LB,Hy	LB, Hy	MS_Hy _(p < 0.001) BL_Hy _(p < 0.001) score_LB _(p < 0.01)

s10724503^1^	34,315,708	LB	LB	group_LB _(p < 0.001) number of spots_LB _p < 0.01) size_LB _(p < 0.001)

rs14763225^2^	34,275,496	LB, Hy		

miRNA-1556	34,315,890	LB	LB	group_LB _(p < 0.002) number of spots_LB _(p < 0.002) size_LB _(p < 0.002)

rs16767662	34,996,069			

rs16110443	36,236,398			

rs14764985	36,533,455	LB	LB	group_LB _(p < 0.03) number of spot_LB _(p < 0.02)

rs16111109	36,959,973			

rs14766124	38,170,684	LB		

rs16132985	41,098,862	LB		

Markers (their informativity and relative genomic positions) in each population can be found in the 3rd column (LB = Lohmann Brown, Hy = Hy-Line). The population and trait showing the association (corresponding p-values in parenthesis) are indicated in the 4^th ^and 5^th ^columns.

^1 ^ZO-2 snupe

^2 ^not mapped in WASHUC2, position based on Groenen et al. 2009 [24].

## Discussion and conclusions

There have been no reports so far of QTL for blood and meat inclusions in eggs of laying hens. This paper describes the first results of detailed analyses of the internal inclusion traits in chickens. QTL were first localized through linkage analysis in a genome scan. This analysis revealed one highly significant QTL on chromosome Z and three suggestive QTLs on chromosomes 1, 2 and 4. The QTL on chromosome Z was fine-mapped and validated with a confirmation study in two independent commercial populations. The association of markers with the trait in these independent samples supported the location of the QTL on chromosome Z. Markers MCW241 and rs14761267 showed most congruent association to inclusion traits in the two tested commercial populations. Thus these markers could be considered as having the best potential for selecting against internal inclusions.

The correct definition of inclusion phenotype is usually demanding if eggs are not studied soon after laying. Sometimes the two types can have similar appearance (i.e. dilution of blood spots to paler meat spots). Combining meat and blood spots to a single variable is a common procedure in breeding companies. However, in QTL mapping this may introduce a bias, especially when one of the components is weighted more than the other variable. According to our results, blood and meat spots are separate entities. The origin of blood spots is red cells, and meat spots are cell masses of epithelium. Distinct sources of the two spot types are supported by their separate locations within the egg. Similar conclusions can be found from [15] or [16]. Campo et al. [6] suggested that internal inclusions are related to shell pigmentation. In another study, a protoporphyrin mutant that reduced shell color significantly also showed a reduction in meat spots [17]. However, according to our results, there are no signs of pigmented cells in the inclusions.

Our results from the F_2 _and LB data (treating blood and meat spots as one trait, with weight on blood spots), might be pulled towards loci affecting blood spots instead of meat spots. Yet, the results in Hy-Line, where those two traits are treated separately, are indicating that the same chromosomal area has an impact on both spot types.

Numerous identified genes can be found from the Ensembl genome database in the QTL region. *ZO-2 *was one of these genes. It was chosen for further studies because of its known biological function together with co-location of microsatellite marker MCW241. *ZO-2 *belongs to the *tight junction protein *family, which is involved in the organization of epithelial and endothelial intercellular junctions. It has an important role in barrier formation. Blood spots in the eggs may be due to the fragility of blood vessel walls in the ovaries.

Symptoms like clotting and bleeding are caused by a genetic defect called familial hypercholanemia (FHAC, OMIM ID#607748) in humans [18], which is caused by a mutation in the *tight junction protein ZO-2*. Compared to our sequencing findings, the mutation in humans is located elsewhere on the *ZO-2 *gene.

The role of the microRNA identified inside the *ZO-2 *gene intron 18 is also interesting. Some reports have demonstrated that miRNAs are transcriptionally linked to the expression of their host genes and processed from the same primary transcript [19]. Thus in addition to regulating its own target genes, gga-mir-1556 might also affect the expression of *ZO-2*.

Target prediction and functional annotations search gave 80 predicted targets for *gga*-*mir*-*1556 *(http://mirdb.org/). Among the target genes is *AGT, angiotensinogen *(located on GGA3 at 42.30 Mb). Angiotensinogen is a precursor of angiotensin, which increases blood pressure. The action of this hypothetical gene would be compatible with the findings of Fry et al. [20] who demonstrated that blood pressure is a factor in susceptibility to blood spot incidence.

According to eQTL studies, a polymorphism explaining gene expression variance may be located either near the gene (cis-eQTL) or further apart on the genome (trans-eQTL)[21]. It has been proposed that trans-eQTL have smaller phenotypic effects than cis-eQTL. In this study we found polymorphism in the miRNA stem-loop that may affect its binding affinities to target genes. Thus the effect could be similar to a trans-acting eQTL. This is supported by the fact that although the effect of the QTL was very significant, it was quite small, approximately 2% of the phenotypic variance.

The heritability of internal inclusions is high enough for the trait to be influenced by conventional breeding [11], but it has not been possible to completely eliminate it by means of selection. We have identified one of the genomic areas influencing the incidence of blood and meat inclusions. In addition, we have confirmed this association in two commercial breeding populations. The actual causative gene or regulatory mechanism still remains unknown. Further investigations are needed before decisions can be made on using the results in marker-assisted selection. The genetic gain is relative to the magnitude of the effect of the locus, and in this case, the effect of the QTL is moderate. However, combining the allele information with conventional selection schemes would yield more information for use in selection decisions.

## Methods

### Mapping populations and genotyping

Three independent egg layer chicken populations were used for different stages in this study. Mapping was done in a two-step approach; first a subset of the F_2 _population was used to a sparse genome scan and thereafter the entire mapping population was used in a consequent fine-mapping step with denser marker map of the most significant QTL region. Two independent pure commercial lines, Lohmann Brown (LB) and Hy-Line (Hy) were used for confirming the QTL result with association analysis. The phases of the study are illustrated in Table 4.

**Table 4:** Work flow

Study	Aim	Material	Method	details
Genome scan	Search for QTL	Sub data of F_2 _population (randomly selected 7 half-sib families)	Linkage analyses of 162 microsatellites on 27 chromosomes	668 F_2 _hens

Fine-mapping	Focusing on the QTL area	Whole F_2 _mapping population (17 half-sib families)	6 new microsatellite markers + 5 SNPs	1599 F_2 _hens

Sequencing	SNP detection	Lohmann Brown, Hy-Line		106 indivuals in LB 20 invidivuals in Hy

Histo-pathology	Tissue analysis	Eggs from a broiler breeding hatchery	Light microscopy study	480 eggs

Confirmation	Association studies in independent commercial lines	1) Lohmann Brown (767 hens)	Mixed models with DMU	whole pure line hen population (767 hens): MCW241 -sub data I: ZO-2 (516 hens) -sub data II: phenotypically extreme 416 hens: panel of 15 SNPs
		2) Hy-Line (290 males)	Linear model with least squares, SAS	-all genotyped with MCW241 -all genotyped with a panel of 5 informative SNPs

Different phases and aims of the study are shown in the 1^st ^and 2^nd ^columns. Populations and subsets are presented in the 3^rd ^column followed with the methods and details in the 4^th ^and 5^th ^columns.

#### F_2 _cross

For the genome scan, an F_2 _cross between two commercial brown pure breeding lines from Lohmann Tierzucht, Rhode Island Red and White Rock, described earlier in Tuiskula-Haavisto et al. [22] was used. The entire mapping population consisted of a total of 1783 individuals, including 30 grandparental males, 47 grandparental females, 16 F_1 _males, 90 F_1 _females, and 1599 F_2 _hens. Rearing and management practices were similar to those in the previous QTL study, see [23].

#### Commercial lines

The Lohmann Brown population consisted of 767 purebred hens from paternal half-sib families with an average of 9.7 offspring per family. Different numbers of individuals were included in analyses depending on the marker used (see Table 4). In the LB, 17 SNP markers, a miRNA polymorphism and one microsatellite marker (MCW241) were genotyped.

The Hy-Line population included a total of 290 males belonging to paternal half-sib families (3,5 males per family). Phenotypes represented sire-daughter averages. The Hy-Line population was analyzed with a microsatellite marker (MCW241) and 4 informative SNP markers within the QTL area (rs14761341, rs14761267, rs14761196 and rs14763225).

#### Genotyping

DNA preparation and genotyping of microsatellite markers have been described in [22]. The number of genotyped individuals in each population is shown in Table 4. For fine-mapping, we selected a set of SNP markers [24] from the QTL region (Table 3). Illumina BeadXpress (http://www.illumina.com) reader was used for genotyping multiplex SNPs (OPA), which were clustered with BeadStudio. The miRNA genotypes were typed by sequencing, and the SNP in the candidate gene by minisequencing (ZO-2 snupe), according to protocols in [25].

#### Phenotypes

Blood and meat spot phenotypes were collected from three consecutive eggs for each hen between the ages of 35 and 41 weeks (Table 5). In contrast to other types of blood spot studies, the hens were fed with adequate feed; no challenge diet was used. Eggs were broken onto a glass sheet to detect the spots. In the F_2 _genome scan, blood and meat spot phenotypes were treated as one trait (BMS_F2_). Eggs were scored based on the presence and size of inclusions ("0" no inclusions, "1" and "2" small or big meat spot, "3", "4" and "5" small, middle-sized or large blood spot). The numeric value of BMS_F2 _was elicited by a transformation, where the average of three eggs was multiplied with 100; thereafter the 'BMS _F2 _unit' referred to a combination of number and severity of the inclusions.

**Table 5:** The populations and phenotypes used in the study

**Pop**.	N	Trait	Scaling	Age of evaluation	Correction
**F_2_**	**1599**	Blood and meat spots (BMS _F2_)	Adjusted from number and type of spots	35, 40, 50 weeks	Average of three sequential eggs, multiplied by 100.

**LB**	**416^1^**	Score	continuous scaling from 0 to 5	36, 40, 42 weeks	LN correction

		Group	count of spots = > high or low	36, 40, 42 weeks	effect of hatch and tier of the battery

		Number of spots	absolute number of spots	36, 40, 42 weeks	(number_corr+0,05)*100

		Size of spots	continuous (in mm)	36, 40, 42 weeks	(size_corr+0,1)*100

		Combination	number * size	36, 40, 42 weeks	(combination_corr+0,1)*100

**Hy-Line**	**290**	Blood spots (BL)	Semi-quantitative	early, late	The phenotypes were expressed as sire-daughter averages

		Meat spots (MS)	Semi-quantitative	early, late	The phenotypes were expressed as sire-daughter averages

The studied populations and the number of individuals in each of them are indicated in the 1^st ^and 2^nd ^columns. The traits are described in the 3^rd ^and 4^th ^columns. The age at time of evaluation is given in the 5^th ^column. The last column presents the correction functions used for normalizing the phenotypic data (LN = natural logarithm).

^1 ^The number of hens genotyped in the Lohmann Brown population varied according to marker: MCW241, 767 hens; ZO-2 SNP,516; 15 other SNPs on the Z chromosome, 416; *mirRNA-1556 *sequencing, 90.

Phenotypes were studied in more detail in the confirmation study of the commercial lines. Blood and meat spots were scored at 36, 40 and 42 weeks of age. For each hen and measurement 3 consecutive eggs were collected and the spots were subjectively scored.

In the Lohmann Brown, the phenotype was recorded as five phenotypic variables of blood and meat spot (Table 5). First, SCORING: scoring scheme was as follows:

0 = no spots

1 = low number of small meat spots

2 = higher number of small meat spots or small number of medium sized meat spots

3 = high number and/or large sized meat spots

4 = low number of small blood spots

5 = higher number of small and/or large blood spots

Prior to QTL analyses the phenotypic mean of the 3 eggs from one hen for every age were corrected for the effect of hatch and position of the hen (tier of the battery). This environmental influence was estimated from a genetic model including the additive genetic effects of the animals. This analysis was done with the Software PEST [26].

where y_ij _= phenotypic observation

AHT_i _= fixed effect of Age-Class, Hatch and Tier i (combined in a multicode)

a_j _= additive genetic effect of animal j

e_ij _= residual

The corrected averages for every hen for the 3 observations at different age were then aggregated into one observation by arithmetic mean. Second, GROUP: based on the scoring, hens were divided into high (spots) or low (no spots). Third, NUMBER of spots. Fourth, SIZE of spots: diameter of the spots (in mm). Fifth, COMBINATION: the function of number and size of spots. The functions used for normalizing the phenotypic data are presented in Table 5.

In the Hy-Line population, eggs were processed within 24 hours of production at a centralized egg quality lab facility. Blood and meat inclusions were identified and treated as two separate traits. Both traits were measured in a semi-quantitative scale, using a score based on the presence and size of the inclusion (0 to 5: "0" if an inclusion was absent, to "5" if an approximately 5 mm inclusion was present). Phenotypes for males were expressed as sire-daughter averages. Descriptive statistics, such as distribution and genetic parameters in the parental lines of F_2 _mapping population is presented in the additional file, Tables S1-S3.

#### Histopathological studies

In order to examine the source of the inclusions, a histopathological study was conducted. Material for the histopathological study was collected from a total of 480 randomly selected eggs from a broiler-breeding hatchery. Fresh, distinct spots were fixed in 10% buffered formalin, routinely processed, embedded in paraffin and serially sectioned at 4 μm. The sections were stained with haematoxylin and eosin (H&E) and studied by light microscopy.

#### QTL mapping

The mapping was done in two stages. At first, a genome scan with 162 microsatellite markers on 27 chromosomes was conducted in a subset of seven half-sib families with 668 F_2 _individuals. Then the entire mapping population including 1599 F_2 _hens was used in the fine-mapping. A new linkage map for the Z-chromosome was calculated by CRI-MAP [27], including 9 microsatellite markers and 5 SNPs. QTL mapping was based on regression analysis using multiple marker information. Autosomes were analyzed with GridQTL [28] and the Z-chromosome by a custom-made program as described in [22,23]. From the Z-chromosome, only the additive effect could be estimated. The empirical significance levels were determined by a permutation test, also described in [22,23].

#### Candidate gene sequencing

A candidate gene was chosen based on its known function and the genetic map information obtained from QTL mapping. The *tight junction protein 2 *gene, *TJP2*, also known as *ZO-2*, was partially sequenced for a single genomic fragment, including two previously reported SNPs (rs10724503 and rs16767170) http://www.ensembl.org/Gallus_gallus/. This area of 546 base pairs contains exon 18 and part of the following intron (genomic location of 34,315,438 - 34,315,986). The primer pair to amplify the fragment was designed with Primer3 http://frodo.wi.mit.edu/primer3/) (forward primer: 5'- AAGCTGCTTCGAAAAATGGA and reverse 5'-GTCACTTGGCAACACAAGGA). This primer pair was also used for sequencing. The sequencing and minisequencing protocols were conducted as described in [25]. For the minisequencing, primers to fragment amplification were forward 5'-CTGTACCGGCAGAACACTGA and reverse 5'- GAAGACACAGTTACTTCCCCTGA. The oligo for minisequencing was 5'- AACCCAGACAGTAAGCAGGG.

#### Fine-mapping

Based on the result from the genome scan, nine more families were included in the QTL analysis. The full population was genotyped with a marker panel of nine microsatellite markers and five single nucleotide polymorphisms (SNPs) chosen from Groenen et al. [24] for the QTL area on chromosome Z. The initial genome scan was conducted with 5 microsatellite markers (ADL117-22cM-MCW331-12 cM-MCW55-16cM-MCW258-38cM-MCW241). Markers used in the fine-mapping were: MCW331-15cM-MCW258-28cM-LEI171-4cM-ADL201-3cM-rs14761196-2cM-MCW241-4cM-rs16767662-7cM-rs16110443-1cM- rs1611109-2cM-rs16132985-5cM-LEI111-1cM-LEI144-2cM-LEI121-17cM-LEI75.

#### Confirmation with association

To confirm the fine-mapping QTL result for blood and meat spots, association was tested in two independent commercial pure lines with more detailed phenotypic traits. Different sets of SNP markers were used depending on their information content in the respective population. In the Lohmann Brown (LB) population, hens were genotyped for the microsatellite MCW241 and minisequenced for a candidate gene SNP (rs10724503) (called herein *ZO-2 *snupe). Additional analyses with 15 SNPs were done with a subset of phenotypically extreme hens from the LB (Table 3). From Hy-Line, a White Plymouth Rock pure line, 290 sires were genotyped with one microsatellite marker (MCW241) and 5 SNP markers from the QTL area. The marker associations with different inclusion traits were conducted separately for each marker (Table 5). The associations in the LB population were estimated with the software package DMU [29], which enabled exploitation of a mixed linear model and inclusion of population structure.

In the Hy-Line population, the marker-trait association was analysed using a linear model with the method of least squares in SAS [30]. Depending on the trait tested T-test, Wilcoxon Rank Sum, or Fisher's exact test was implemented to find association between phenotypic traits and markers.

## Authors' contributions

MH designed and performed the genotyping and sequencing work, participated in the statistical analysis (linkage analyses) and wrote the manuscript. MT-H contributed to the study design and data collection and analyses. VA and PU contributed to the statistical analyses (association analyses). PV did the histopathological study. MS, DC, JA, NOS, JF and RP contributed to the study design, provided phenotypic data and animal samples. JV supervised the study and edited the manuscript. All authors read and approved the final manuscript*.*

## Supplementary Material

Additional file 1 **Table S1: Estimates of Genetic Parameters for Inclusions in pure lines (grandparental lines) of Lohmann Brown (Heritability on the diagonal and genetic correlation of the off-diagonal)**. **Table S2: **Distribution of phenotypes in the grandparental lines of Lohmann Brown for the subjective combined score (3 eggs per hen). **Table S3: **Distributions of phenotypes in the grandparental lines of Lohmann Brown for number and size of the spots (only for Rhode Island Red line). Click here for file
