# Supplementary material for: Mapping of QTL affecting incidence of blood and meat inclusions in egg layers
Source: BMC Genet. 2011 Jun 13;12:55. doi: 10.1186/1471-2156-12-55 (PMC3145579; doi:10.1186/1471-2156-12-55)
Supplement: Additional file 1 — Table S1: Estimates of Genetic Parameters for Inclusions in pure lines (grandparental lines) of Lohmann Brown (Heritability on the diagonal and genetic correlation of the off-diagonal). Table S2: Distribution of phenotypes in the grandparental lines of Lohmann Brown for the subjective combined score (3 eggs per hen). Table S3: Distributions of phenotypes in the grandparental lines of Lohmann Brown for number and size of the spots (only for Rhode Island Red line). [file 1471-2156-12-55-S1.DOC]

# Additional files (Tables S1, S2 and S3). Distribution and genetic parameters for Inclusions in the grandparental lines of Lohmann Brown

## Table S1: Estimates of Genetic Parameters for Inclusions in pure lines (grandparental lines) of Lohmann Brown (Heritability on the diagonal and genetic correlation of the offdiagonal)

Line 1 (Rhode Island Red)

|  | Score | Blood spots (number*size) | Meat spot (number*size) |
| --- | --- | --- | --- |
| Score | 0,05 | -0,90 | -0,70 |
| Blood spots |  | 0,04 | 0,83 |
| Meat spots |  |  | 0,01 |

Line 2 (White Rock)

Heritability for Score: h2=0,01

**Table S2:** Distribution of phenotypes in the grandparental lines of Lohmann Brown for the subjective combined score (3 eggs per hen)

| Score |  | Rhode Island Red  (n=4355 hens) | White Rock  (n=4402 hens) |
| --- | --- | --- | --- |
| 0 | no spots | 75.0 % | 83.7 % |
| 1 | low number small meat spots | 4.5 % | 7.3 % |
| 2 | higher number small meat spots | 2.5 % | 4.9 % |
| 3 | large meat spots | 3.1 % | 2.1 % |
| 4 | low number small blood spots | 13.6 % | 1.8 % |
| 5 | higher number and/or large blood spots | 1.3 % | 0.2 % |

***Table S3:*** Distribution of phenotypes in the grandparental lines of Lohmann Brown for number and size of the spots (only for Rhode Island Red line)

| Blood Spots | | | | Meat Spots | | | |
| --- | --- | --- | --- | --- | --- | --- | --- |
| number | frequency | size | frequency | number | frequency | size | frequency |
| 0 | 58.2% | 0 | 58.2% | 0 | 88.1% | 0 | 88.1% |
| 1 | 15.1% | 1 | 17.6% | 1 | 7.9% | 1 | 3.4% |
| 2 | 6.8% | 2 | 9.6% | 2 | 1.1% | 2 | 2.8% |
| 3 | 7.6% | 3 | 5.9% | 3 | 0.5% | 3 | 1.8% |
| 4 | 2.5% | 4 | 4.0% | 4 | 0.1% | 4 | 1.1% |
| 5 | 3.9% | 5 | 0.3% | 5 | 0.1% | 5 | 0.7% |
| >5 | 5.9% | >5 mm | 4.4% | >5 | 2.2% | >5 | 2.1% |

The number and size of the spot was not recorded from the same eggs (but from the same hens) therefore the subjective score and number/size recording is influenced from different recording time.
